# Exosome-mediated crosstalk between epithelial cells amplifies the cell injury cascade in CaOx stone formation

**DOI:** 10.1186/s13036-023-00324-0

**Published:** 2023-02-28

**Authors:** Yuanyuan Yang, Senyuan Hong, Qing Wang, Shaogang Wang, Yang Xun

**Affiliations:** 1 Department of Urology, Tongji Hospital, Tongji Medical College, Huazhong University of Science and Technology, Wuhan, 430030 Hubei China; 2 Department of Urology, Guizhou Provincial People’s Hospital, Guizhou University, Guiyang, 550000 Guizhou China; 3 Department of Research Laboratory Center, Guizhou Provincial People’s Hospital, Guizhou University, Guiyang, 550000 Guizhou China

**Keywords:** CaOx stones, Exosomes, Intercellular crosstalk, Oxidative stress, *MAPK/P38* pathway

## Abstract

**Background:**

Calcium oxalate (CaOx) stone disease is found worldwide. To explore the role of exosomes as a mediator of intercellular crosstalk during CaOx stone formation, we conducted this study, which may provide a new insight into the treatment and prevention of CaOx stones.

**Methods:**

Exosomes derived from HK2 cells with (EXO(S)) or without (EXO(C))CaOx crystal stimulation were cocultured with normal tubular epithelial cells and subcapsularly injected into rat kidneys. Then, oxidative stress levels, the *MAPK* signalling pathway and osteogenic changes were detected via qPCR, Western blotting, immunofluorescence and immunohistochemical staining. In vivo fluorescence imaging and exosome internalization assays showed the absorption and utilization of exosomes.

**Results:**

EXO(S) increased the reactive oxygen species (ROS) level and activated the expression of *BMP2, OPN* and *OCN* via the *MAPK/P-38* pathway both in vivo and in vitro. In vivo experiments showed that preinjection of EXO(S) aggravated, while preinjection of EXO(C) ameliorated, these effects. Crystal depositions were significantly increased in SD rats injected with GAM when they were preinjected with EXO(S), and these effects could be reversed after preinjection with EXO(C).

**Conclusion:**

Our study revealed that exosome-mediated intercellular crosstalk could accelerate the formation of CaOx stones by promoting oxidative stress and the osteogenic cascade in normal tubular epithelial cells.

**Graphical Abstract:**

HK2 cells stimulated with CaOx crystals released more exosomal *miR-223-3p* and *S100A8* comparing with normal HK2 cells. These exosomes derived from HK2 cells stimulated with CaOx (EXO(S)) could amplify the oxidative stress and osteogenic changes via *MAPK/P-38* pathway, which finally led to the formation of Randall’s plaque.

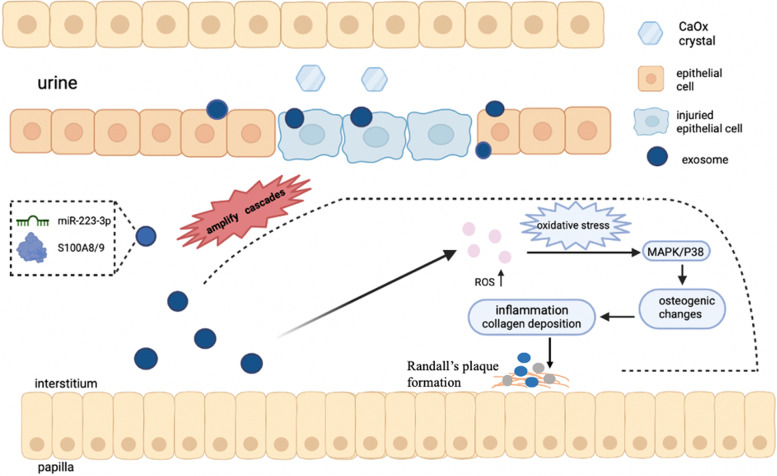

**Supplementary Information:**

The online version contains supplementary material available at 10.1186/s13036-023-00324-0.

## Introduction

Kidney stones are a global disease with a prevalence of 5% ~ 15% [1]. Moreover, kidney stones have a high recurrence rate of 50% within 10 years. The high incidence and recurrence rates make kidney stones a major health burden both clinically and economically. The surgical treatments of kidney stones have substantially improved due to the development of minimally invasive technology. However, the prevention and medical treatment of kidney stones are still insufficient because mechanism of stone formation remains unclear [2].

CaOx stones comprise 80% of kidney stones and are the most common stone type [3]. Although the mechanism of stone formation has not been fully elucidated, many theories have been proposed, and Randall’s plaque involves a classical theory. Randall’s plaque is an ectopic calcification located in the renal papillary interstitium and may originate from the basement membrane of small tubules in the thin segment of the Henle loop, followed by gradual progression subcutaneously to the interstitium and urinary tract, finally breaking through the urinary tract epithelium, contacting urine and becoming the attachment site of crystals [4]. Sarah A. Howles et al. have found that crystals could cause injury to epithelial cells through physical contact, toxicity and receptor activation. These processes might induce osteogenic changes and promote the production of molecules modulating inflammation and mineralization, ultimately leading to Randall’s plaques [3]. Our previous studies have demonstrated that hypercalciuria and hyperoxaluria could promote osteogenic changes and the production of inflammatory cytokines in HK2 cells via the *NOX4/MAPK/P-38* axis, and finally lead to Randall’s plaques [5]. However, clinically, many patients with CaOx stones do not have hypercalciuria and hyperoxaluria or they have transient hypercalciuria and hyperoxaluria [6]. Therefore, we hypothesized that another factor could prolong the duration of the effects and extend the range of the cells it affects.

Recently, intercellular crosstalk has been confirmed to be a key modulator in the process of many diseases,and extracellular vesicles are important mediators of intercellular crosstalk [7]. Previous studies have indicated that vesicles derived from cells might play key roles in stone formation [8]. Exosomes are vesicles with a diameter of 30-150 nm. Exosomes are generally stored in intraluminal vesicles and can be released when fusing with the cell wall. Moreover, exosomes can avoid endosomal-lysosomal degradation, which make it easier for them to affect distant cells and amplify these effects [9]. Exosomes have been found to play important roles in many diseases [10]. Moreover, exosomes have been applied in the treatment of many diseases [11, 12]. In this study, we explored the relationship between exosomes derived from tubular epithelial cells and Randall’s plaque development and confirmed the important role of exosome-mediated intercellular crosstalk in the process of CaOx stone formation, which might provide a new insight into the prevention and treatment of the kidney stone disease.

## Materials & methods

### Animal experimental design

Thirty male SD rats (8 weeks old,180–220 g) were purchased from the Experimental Animal Center of Tongji Hospital, Tongji Medical College, Huazhong University of Science and Technology. The rats were acclimatized to a 12 h light/dark cycle in a specific pathogen-free animal house for 1 week before the start of the experiments. The rats were divided into six groups with 5 rats each group: the control group: normal feeding without any treatments; the GAM group: intraperitoneal injection of GAM (glyoxylic acid monohydrate) at 80 mg/kg/day for 9 days; the EXO(C) group: renal subcapsular injection of EXO(C) at 40 mg/day every week for 2 weeks; the EXO(S) group: renal subcapsular injection of EXO(S) at 40 mg/day every week for 2 weeks; renal subcapsular injection of normal saline (NS) was conducted in these two groups on the other side of the rat kidney as the sham group. For the EXO(C) + GAM group, pre-renal subcapsular injection with EXO(C) was performed every week for 2 weeks, followed by intraperitoneal injection of GAM at 80 mg/kg/day for 9 days; for the EXO(S) + GAM group, prerenal subcapsular injection with EXO(S) was performed every week for 2 weeks, followed by intraperitoneal injection of GAM at 80 mg/kg/day for 9 days; renal subcapsular injection of NS was conducted in these two groups on the other side of the rat kidney as the sham group. All procedures were performed following the Animal Management Rules of the Ministry of Health of the People’s Republic of China. The operations were approved by the Animal Care and Use Committee of Tongji Hospital, Tongji Medical College, Huazhong University of Science and Technology (IACUC Number:2746). All rats were euthanised after the above treatments. The rat kidney was ground up with a tissue grinder. Then, the tissue was centrifuged at 3000 rpm for 10 min, and the supernatant was stored for further exploration. The rest kidney tissues were used for histological examination and Von Kossa staining.

### Cell culture and treatment

The HK2 cell line was purchased from Procell (Wuhan, China) and cultured in RPMI 1640 medium (HyClone, USA) containing 10% foetal bovine serum (FBS) (Gibco, Grand Island, NY, USA), which had been depleted of exosomes before being added to the medium. The cells were exposed to CaOx for 24 h at a concentration of 2 mM, and then, the supernatant was collected for isolation of exosomes. Exosomes derived from HK2 cells without CaOx crystal stimulation were defined as EXO(C), Exosomes derived from HK2 cells stimulated with CaOx crystals were defined as EXO(S). EXO(C) and EXO(S) were added to HK2 cells and cocultured for 48 hours.

### Exosome isolation

Exosomes derived from cells were isolated through multistep centrifugation. First, HK2 cells cultured in RPMI 1640 containing exosomes depleted of FBS were centrifuged at 300 g for 15 minutes to eliminate the dead cells and debris. Then, the samples were centrifuged at 2000 g for 30 minutes and 10,000 g for 30 minutes. Each supernatant was filtered in a 100-kDa MWCO ultrafiltration centrifuge tube (Amicon-Ultra, Millipore, USA). Then, the supernatant was centrifuged at 4 °C and 120,000 g for 90 minutes, the supernatant was discarded, and the pellet was resuspended in PBS and centrifuged at 4 °C, 120000 g for 90 minutes again to collect exosomes.

### In vivo fluorescence imaging

Briefly,40 μg of exosomes labelled with PKH26 (UR52302, Umibio, China) were locally injected into the rat kidneys (via methods described previously in the animal design section). Exosome accumulation and dynamics were tracked by in vivo fluorescence imaging on Day 1, 3, 7, 10and 14 via a Lago/Lago X Imager (Lago/Lago, Spectral Instruments Imaging, USA).

### Exosome labelling with Dil and uptake study

The purified exosomes were labelled with a Dil red fluorescent labelling kit (Biodee, Beijing) according to the manufacturer’s instructions. Then, the labelled exosomes were cocultured with HK2 cells. Observations were conducted at 0, 6, and 12 hours after incubation with a laser confocal microscope (SP8, Leica, Germany).

### Transmission Electron microscopy (TEM) and nanoparticle tracking analysis (NTA)

The procedures for conducting transmission electron microscopy and nanoparticle tracking analysis were the same as those described in our previous study [13].

### Measurement of reactive oxygen species (ROS) by dihydroethidium (DHE)

Intracellular ROS production was detected via a Reactive Oxygen Species Assay Kit (Beyotime, S00335). Cells were cultured in a 6-well plate and stimulated by EXO(C) and EXO(S), digested these cells with 0.25% trypsin without EDTA and collected after termination of digestion, washed the cells once with PBS, and set aside. Following the instructions of the ROS detection kit, ROS were detected by flow cytometry (Beckman, CytoFLEX, USA). ROS in renal tissues were measured with DHE fluorescence. Kidney tissues of different groups were ground into homogenate and used for detection of ROS production following the instructions of the ROS detection kit. Then, the fluorescence of DCF was detected via multimode microplate reader (Tecan,M200 PRO).

### Measurement of CAT,SOD and MDA

MDA represents products of lipid peroxidation, and SOD and CAT are key antioxidative biomarkers. The MDA level and SOD and CAT activity were detected by chemiluminescence methods via MDA, CAT, and SOD detection kits (NanJing，JianCheng Bioengineering Institute, China) were used, and detection was conducted following the manufacturer’s instructions. The OD values of MDA were 532 nm, and those of CAT were 405 nm. The OD values at 550 nm were measured for SOD.

### Western blot analysis

Western blotting was performed following the method we used in our previous study [13]. The antibodies included anti-rabbit BMP2 (1:2000, BOSTER, China), anti-rabbit OPN (1:2000, SANTA CRUZ, USA), anti-rabbit OCN (1:2000, SANTA CRUZ, USA), anti-rabbit β-actin antibodies (1:2000, BOSTER, China), anti-mouse TSG101 (1:2000, Abcam, USA), anti-mouse CD9 (1:2000, Abcam, USA), anti-mouse Alix (1:2000, Abcam, USA),anti-mouse Calnexin (1:2000, Abcam, USA), and anti-rabbit and mouse immunoglobulin G (IgG) horseradish peroxidase (HRP)-linked antibody (1:5000, BOSTER, China). All WB quantification was performed using three independent WBs.

### RT-qPCR

Total RNA was extracted from cells with different treatments via TRIzol reagent (Ambion, USA), and reverse transcription reactions were performed with a complementary DNA Synthesis Kit (Yeasen, China). RT-qPCR was conducted via SYBR Green Master Mix (Hieff qPCR SYBR Green Master Mix (High Rox Plus)) on the ABI Prism 7300 system (Thermo Fisher Scientific, USA). The 2 − ΔΔCt method was used to analyse the mRNA expression levels. The primer sequences used in this study are listed in Supplementary Table 1 (Stable1).

### Von Kossa staining

The renal tissues of rats with different treatments were collected and fixed in 10% paraformaldehyde, dehydrated, embedded, sliced and washed them with ddH_2_O for 1 min. Crystal depositions of these renal tissues were then analysed by Von Kossa staining following the manufacturer’s instructions (HEPENGBIO VON KOSSA Kit, China).

### Immunofluorescence (IF) assays and immunohistochemical staining (IHC)

Cells with different treatments were cultured in 6-well plates with slides preset in the wells. The slides were washed for 3 times in PBS,fixed with 4% paraformaldehyde for 15 min, permeabilized with 0.5%Triton X-100 (PBS preparation) for 20 min(room temperature) and blocked with 5% BSA for 30 min. The cells were incubated with OPN and OCN antibodies (1:100) overnight (4 °C), and then incubated with FITC-conjugated goat anti-rabbit IgG (1:100) for 1 h (25 °C). DAPI (4′,6-diamidino-2-phenylindole) was coincubated with cells for 5 min, and then, the fluorescent signals were observed via a BX53 microscope (Olympus, Tokyo, Japan). Immunohistochemical staining was used to detect tissue proteins. Antibodies against BMP2, OPN and OCN (1:100) were coincubated with these section preparations. Images were then observed with a BX53 microscope (Olympus, Tokyo, Japan).

### Statistical analysis

The results are reported as the mean of at least three independent experiments. GraphPad Prism 5 (GraphPad Software, Inc., CA, USA) and SPSS24.0 (SPSS Statistics Inc., Chicago, USA) were used. The statistical methods were selected as we previous described [6]. A * *p* < 0.05 was considered statistically significant.

## Results

### Identification of exosomes derived from HK2 cells

**Fig. 1:**
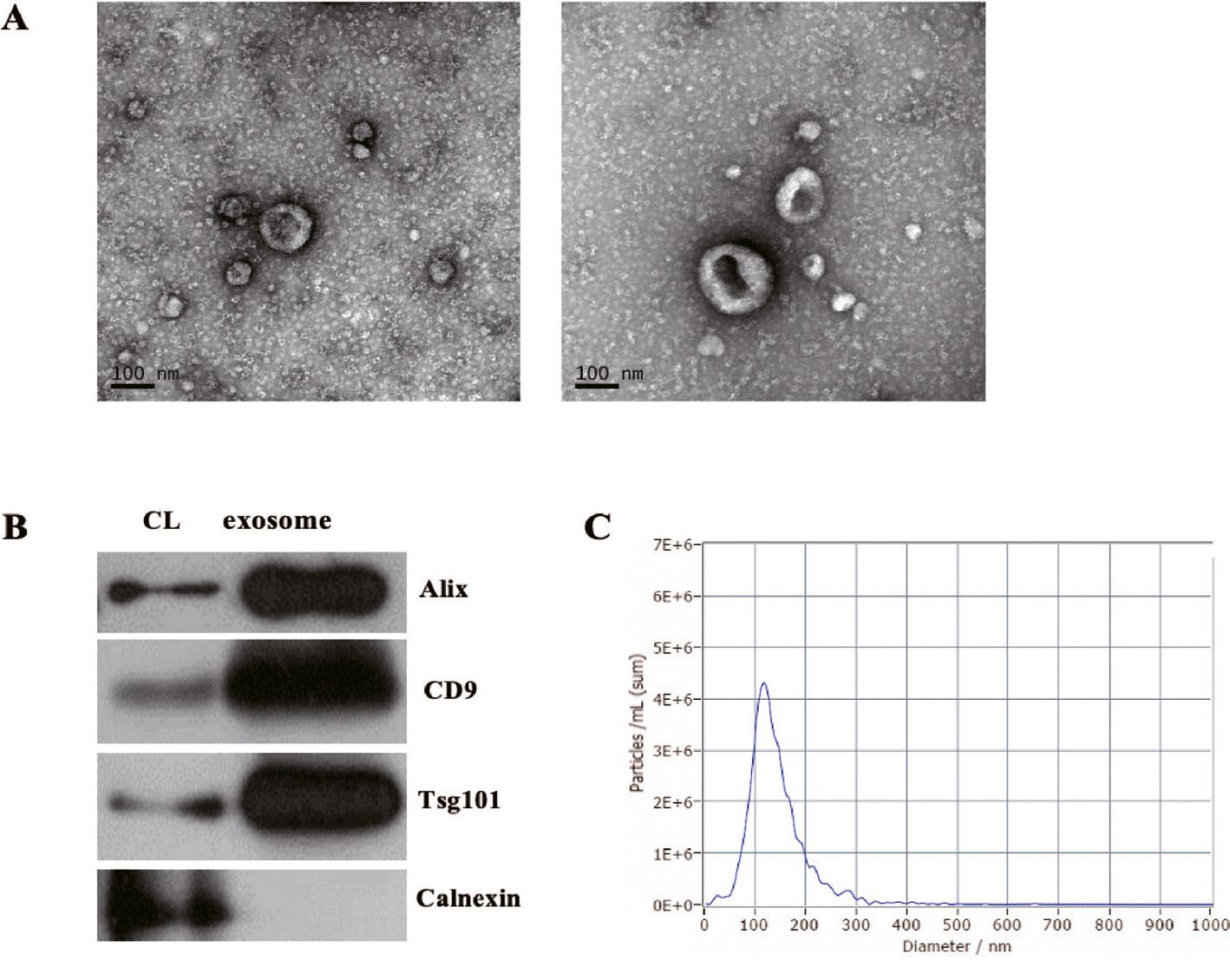
Identification of exosomes derived from HK2 cells. **A** TEM (Transmission Electron Microscope) showed the exosomes isolated from HK2 cells with the typical saucer-like morphology. **B** Western blotting confirmed that *CD9, TSG101* and *Alix* were highly expressed in isolated exosomes while calnexin had lower expression. **C** NTA (Nanoparticle Tracking Analysis) demonstrated that exosomes isolated from HK2 cells were mostly concentrate in 50–200 nm in diameter

Transmission Electron Microscope (TEM) showed that exosomes isolated from HK2 cells had the typical saucer-shaped morphology (Fig. 1A). Western Blot analysis indicated that the expression of *Alix, CD9 and TSG101* was higher in exosomes than in cell lysate (CL), while Calnexin was showed lower expression (Fig. 1B). Nanoparticle Tracking Analysis (NTA) demonstrated that exosomes isolated from HK2 cells were mostly 50-200 nm in diameter, with a peak at 102.1 nm (Fig. 1C).

### Exosomes labelling and uptake assay

**Fig. 2:**
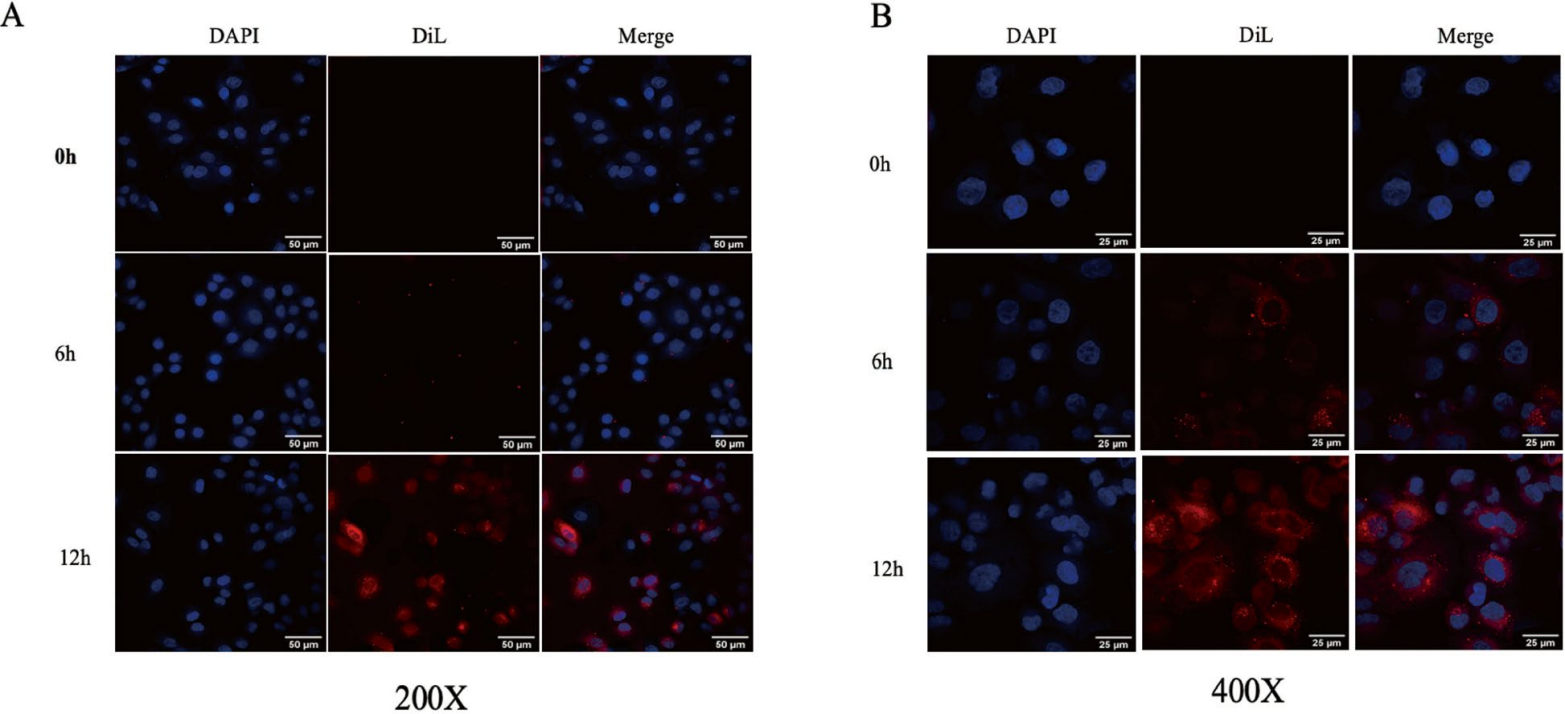
Exosome labelling and uptake assay. Uptake of exosomes by HK2 cells. Representative confocal images of Dil-labelled exosomes taken up by HK2 cells at different culture times are shown. The scale bars are 25 μm and 50 μm

Exosomes derived from HK2 cells were stained with Dil and then added to HK2 cells prestained with DAPI, confocal microscope indicated that HK2 cells took up exosomes derived from other cells overtime. Exosomes were completely absorbed at 12 h (Fig. 2).

### Effects of exosomes on the oxidative stress injury and activation of the MAPK signalling pathway in HK2 cells

**Fig. 3:**
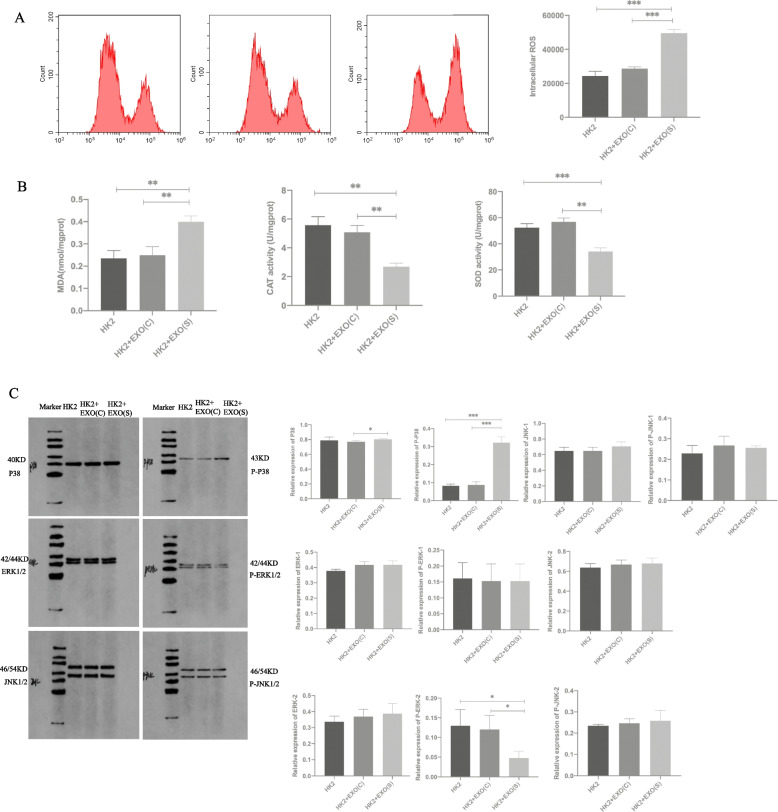
Effects of exosomes on the oxidative stress injury and the activation of the MAPK signalling pathway in HK2 cells. **A** EXO(S) enhanced the ROS level of HK2 cells. **B** MDA, CAT and SOD levels in HK2 cells stimulated with exosomes derived from different conditions. **C** Western blot assays showed the expression levels of the *MAPK* signalling pathway components in the HK2 cells stimulated with EXO(C) or EXO(S). The data are expressed as the mean ± SE. ∗*P* < 0.05 compared with the HK2 group. EXO(C) represents exosomes derived from HK2 cells without CaOx stimulation, while EXO(S) represents exosomes derived from HK2 cells stimulated with CaOx

Intracellular ROS levels indicated that EXO(S) could accelerate the production of ROS in HK2 cells (Fig. 3A). EXO(S) could reduce superoxidative dismutase (SOD) and catalase (CAT) activity and increase malondialdehyde (MDA) levels in HK2 cells,while the stimulation of EXO(C) did not result in any significant difference compared with the control (Fig. 3B). Western Blot analysis showed that the *P-P38* pathway was increased in the HK2 cells stimulated with EXO(S),while *P-ERK* and *P-JNK* showed no significant difference in *P-ERK* and *P-JNK* (Fig. 3C).

### Effects of exosomes on the activation of osteoblastic-associated protein expression in HK2 cells

**Fig. 4:**
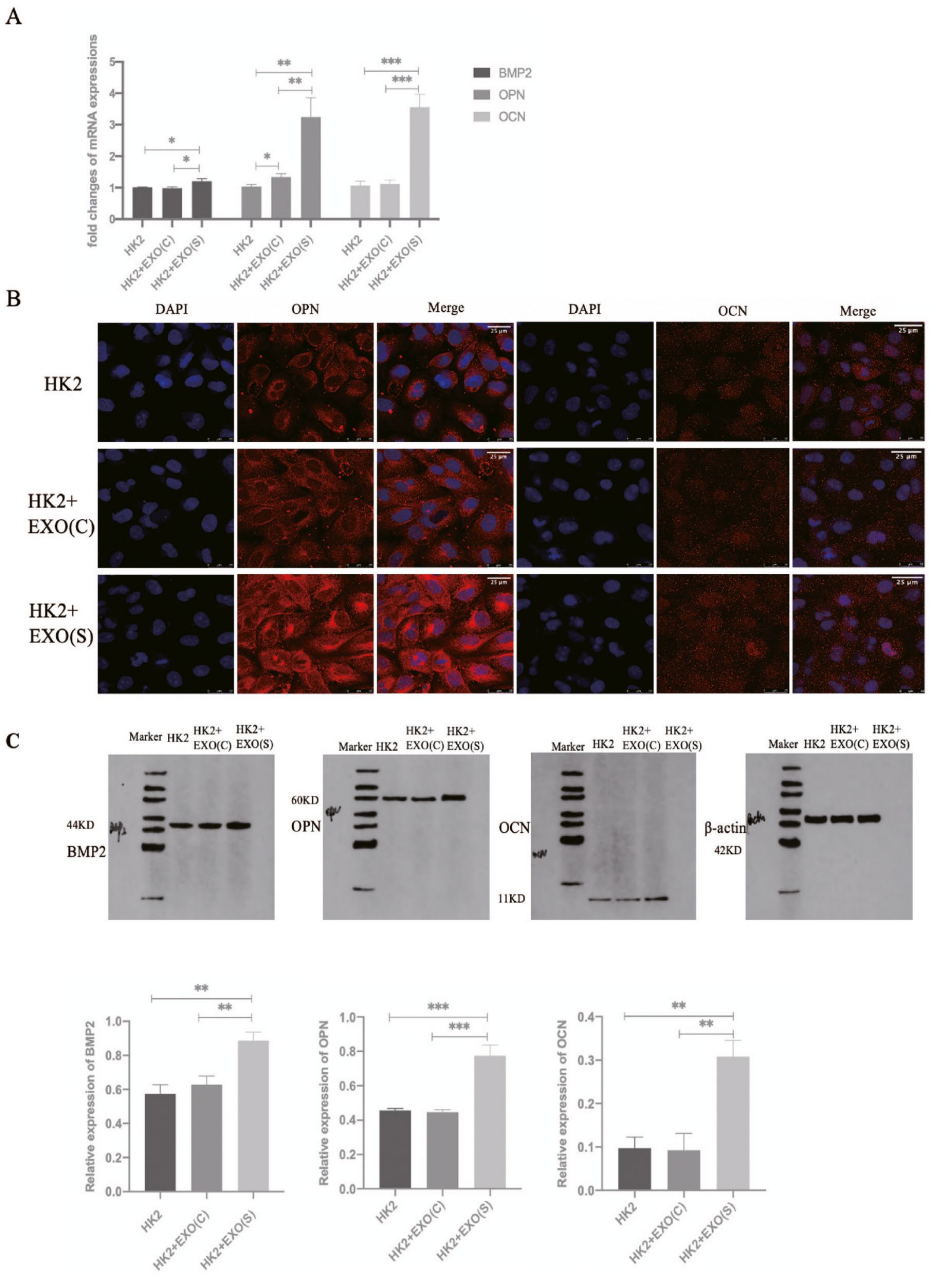
Effects of exosomes on the activation of osteoblastic-associated protein expression in HK2 cells. **A** RT-qPCR analysis of *BMP2,OPN* and *OCN* expression in the HK2 cells stimulated with exosomes derived from HK2 cells under different conditions. **B** Immunofluorescence analysis of *OPN* and *OCN* expression in the HK2 cells stimulated with exosomes derived from HK2 cells under different conditions. **C** Western blot assays showing the expression levels of *BMP2, OPN, and OCN* in the Hk2 cells stimulated with EXO(C) or EXO(S). The data are expressed as the mean ± SE. ∗*P* < 0.05 compared with the HK2 cells

The qPCR results showed that EXO(S) promoted the expression of *BMP2, OPN* and *OCN*, while EXO(C) did not result in significant changes (Fig. 4A). Immunofluorescence analysis indicated that the HK2 cells stimulated with EXO(S) exhibited increased expression of *OPN* and *OCN*. The increase in *OPN* expression was more obvious than that in *OCN* (Fig. 4B). Western Blot analysis demonstrated that the osteoblastic associated proteins *BMP2, OPN* and *OCN* were more highly expressed in HK2 cells stimulated with EXO(S) (Fig. 4C).

### Exosomes derived from HK2 cells were retained in rat kidney for 7 days

**Fig. 5:**
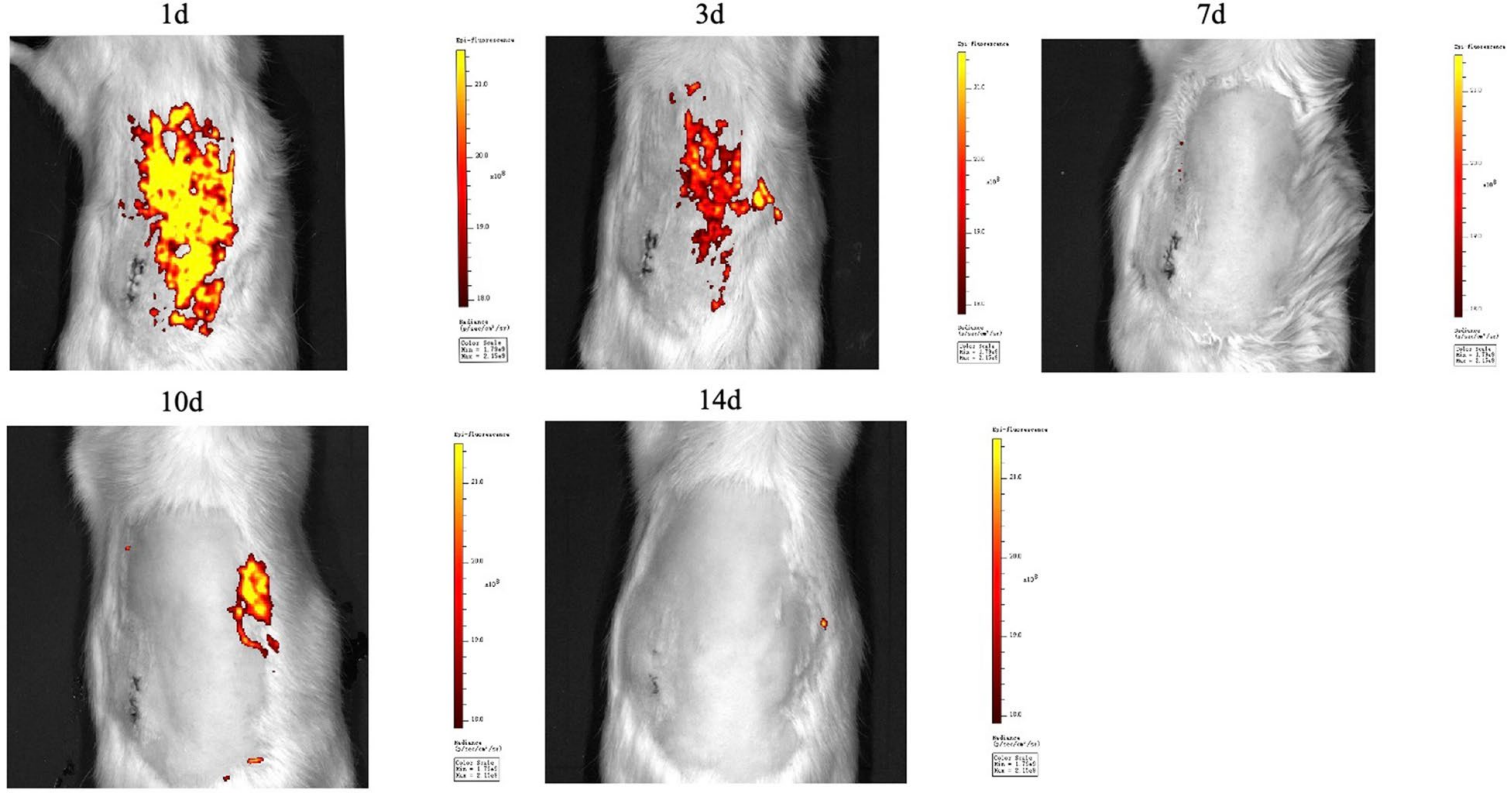
Exosome renal subcapsular injection and in vivo imaging. Representative images of SD rats that underwent renal subcapsular injection with PKH26-labelled exosomes at different time points. The signal intensities of PKH26-labelled exosomes in SD rats after injection are presented as a bar graph

To explore the retention time of exosomes in SD rats after renal subcapsular injection, we dyed the exosomes derived from HK2 cells were dyed with PKH26, and the signal intensities of the exosomes (represented the amount of exosome) at Days 1, 3, 7, 10 and 14 were detected by in vivo fluorescence imaging. The peak signal intensity for exosomes was 24 h after injection, and metabolism was completed by Day 7. Then, we injected exosomes again at Day 7, and the signal intensities were retained until Day 14 (Fig. 5).

### Effects of exosomes derived from HK2 cells under different conditions on oxidative stress injury and activation of the *MAPK* signalling pathway in rat kidneys

**Fig. 6:**
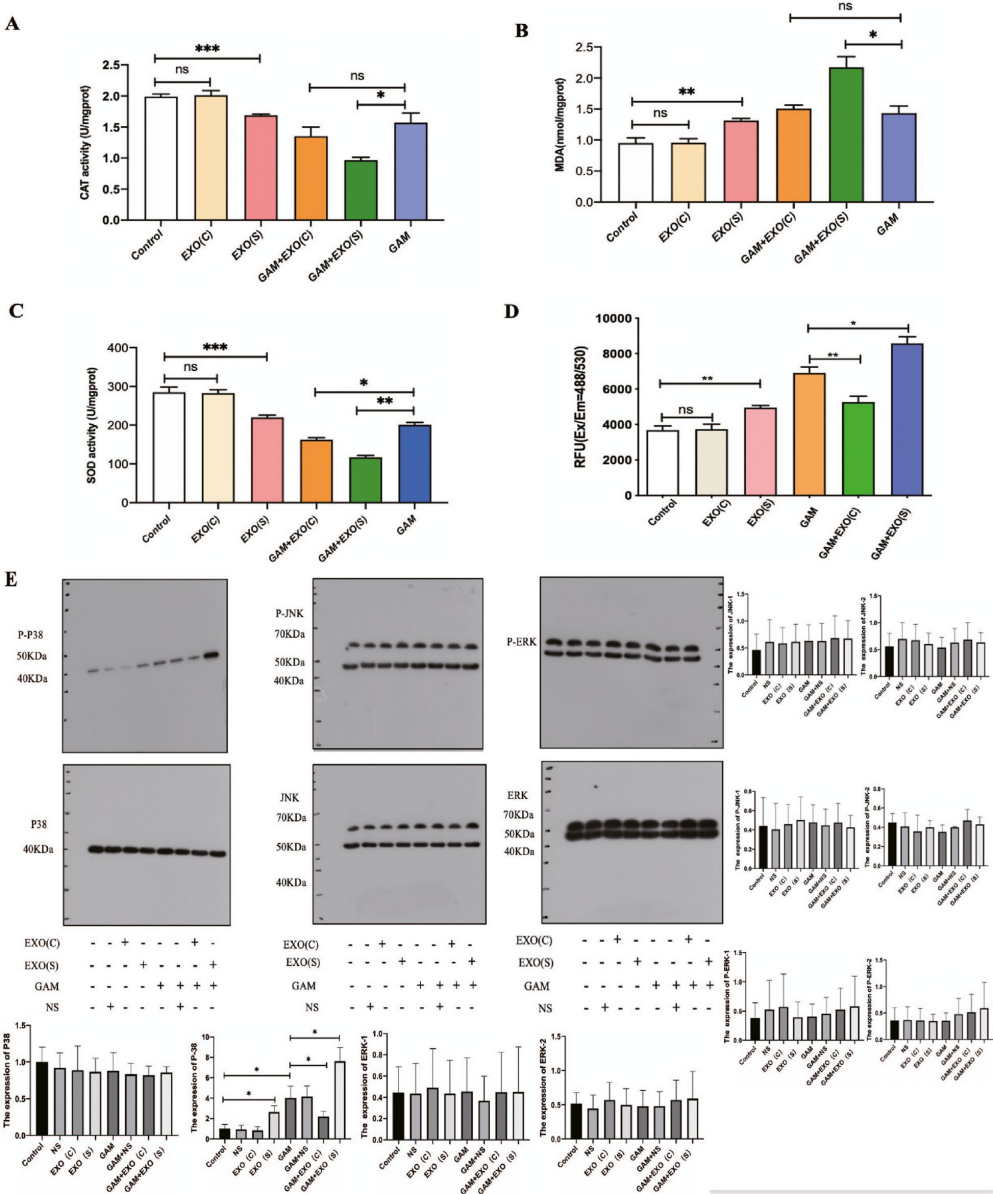
Effects of exosomes derived from HK2 cells under different conditions on oxidative stress injury and the *MAPK* signalling pathway in rat renal tissues with different treatments. **A** Measurement of CAT activity in the SD rats with different treatments. **B** MDA expression in the SD rats under different conditions. **C** SOD activity of the SD rats with different treatments. **D** ROS levels in the SD rats under different conditions. The expression of *P-P38* was increased in the rats injected with EXO(S) or injected with GAM, pre-EXO(C) renal subcapsular injection could ameliorate these effects while pre-EXO(S) renal subcapsular injection could accelerate the expression of *P-P38*. There were no significant changes in the expression of *P-JNK* and *P-ERK*. The data are expressed as the mean ± SE. ∗*P* < 0.05

Renal subcapsular injection of only EXO(S) reduced the CAT activity and SOD activity and increased the level of MDA in SD rats, and only renal subcapsular injection of only EXO(C) did not result in any changes. Moreover, intraperitoneal injection of GAM enhanced the MDA level and reduced CAT and SOD activity in SD rats. Prerenal subcapsular injection of EXO(S) aggravated these effects (Fig. 6A-C). We also presubcapsularly injected NS on the other side of the SD rat kidney for the sham group. We found that prerenal subcapsular injection of NS did not affect the oxidative stress level (Supplementary Fig. 1). Renal subcapsular injection of EXO(C) in rats did not result in any difference, while injection of EXO(S) promoted the ROS production compared with that in the control groups. The SD rats injected with glyoxylic acid exhibited increased ROS level, and preinjection of EXO(C) ameliorated this effect, Preinjection of EXO(S) aggravated this effect (Fig. 6D). Western Blot assays indicated that one of the *MAPK* signalling pathway components, *P-P38*,was increased in the SD rats injected with EXO(S), and injection of GAM also promoted the expression of *P-P38*. Moreover, preinjection of EXO(C) attenuated the effect of GAM, while perinjection of EXO(S) aggravated the effect of GAM. There was no significant difference between the expression of *P-ERK* and *P-JNK*. Injection of NS on the other side of the kidney as a sham control did not cause any changes (Fig. 6E).

### Effects of exosomes derived from HK2 cells under different conditions on the activation of osteoblastic-transmission in rat kidneys

**Fig. 7:**
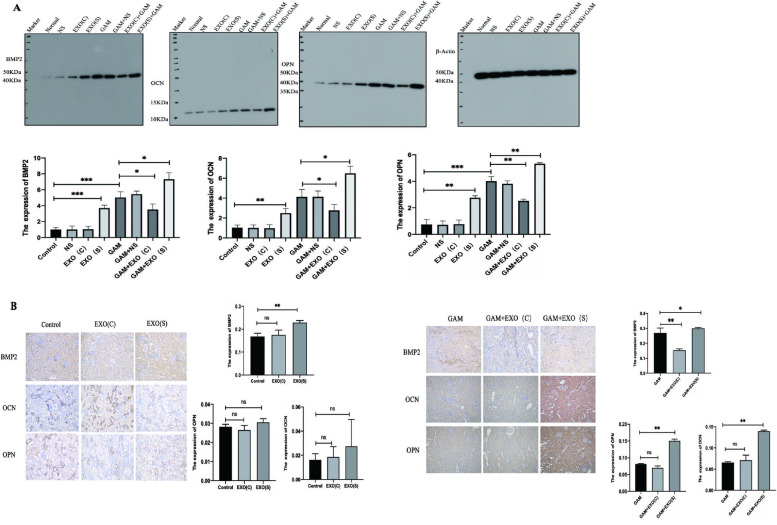
Effects of exosomes derived from HK2 cells under different conditions on the activation of osteoblastic-associated protein expression in rat kidneys. **A** *BMP2,OPN, and OCN* were highly expressed in the rats injected with EXO(S) or GAM, and pre-EXO(C) renal subcapsular injection could ameliorate these effects,while pre-EXO(S) renal subcapsular injection could accelerate the expression of osteoblastic-associated proteins. The expression levels of *BMP2,OPN, and OCN* were quantified via ImageJ. **B** The expression of *BMP2,OPN* and *OCN* in the rats with different treatments (magnification 200X). Three rats from each group were used for quantification via ImageJ. Three animals from each group were used for these data. The data are expressed as the mean ± SE. ∗*P* < 0.05

Western Blot results indicated that the expression of osteoblastic-associated proteins *BMP2,OPN* and *OCN* were promoted in the rats injected with EXO(S), and there were no significant differences between the rats injected with EXO(C) and the control groups. Intraperitoneal injection of GAM promoted the expression of *BMP2,OPN* and *OCN* in SD rats, and preinjection with EXO(C) ameliorated the effects of GAM, while preinjection of EXO(S) aggravated the effects of GAM. Injection of NS on the other side of the kidney of rats injected with exosomes as a sham control resulted in no significant differences. Moreover, immunohistochemical analysis revealed that the expression of osteoblastic-associated protein *BMP2* was higher in the SD rats only injected with EXO(S) compared with control group. It was also obvious increasing in SD rats injected with GAM. Preinjection with EXO(C) reversed this effect, and preinjection with EXO(S) accentuated the effect. The expression of *OPN* and *OCN* did not show any differences in the SD rats injected with EXO(C) and EXO(S). Preinjection with EXO(S) promoted the expression of *OPN* and *OCN* in the SD rats injected with GAM (Fig. 7).

### Effects of exosomes derived from HK2 cells under different conditions on crystal deposition in the rat kidney

**Fig. 8:**
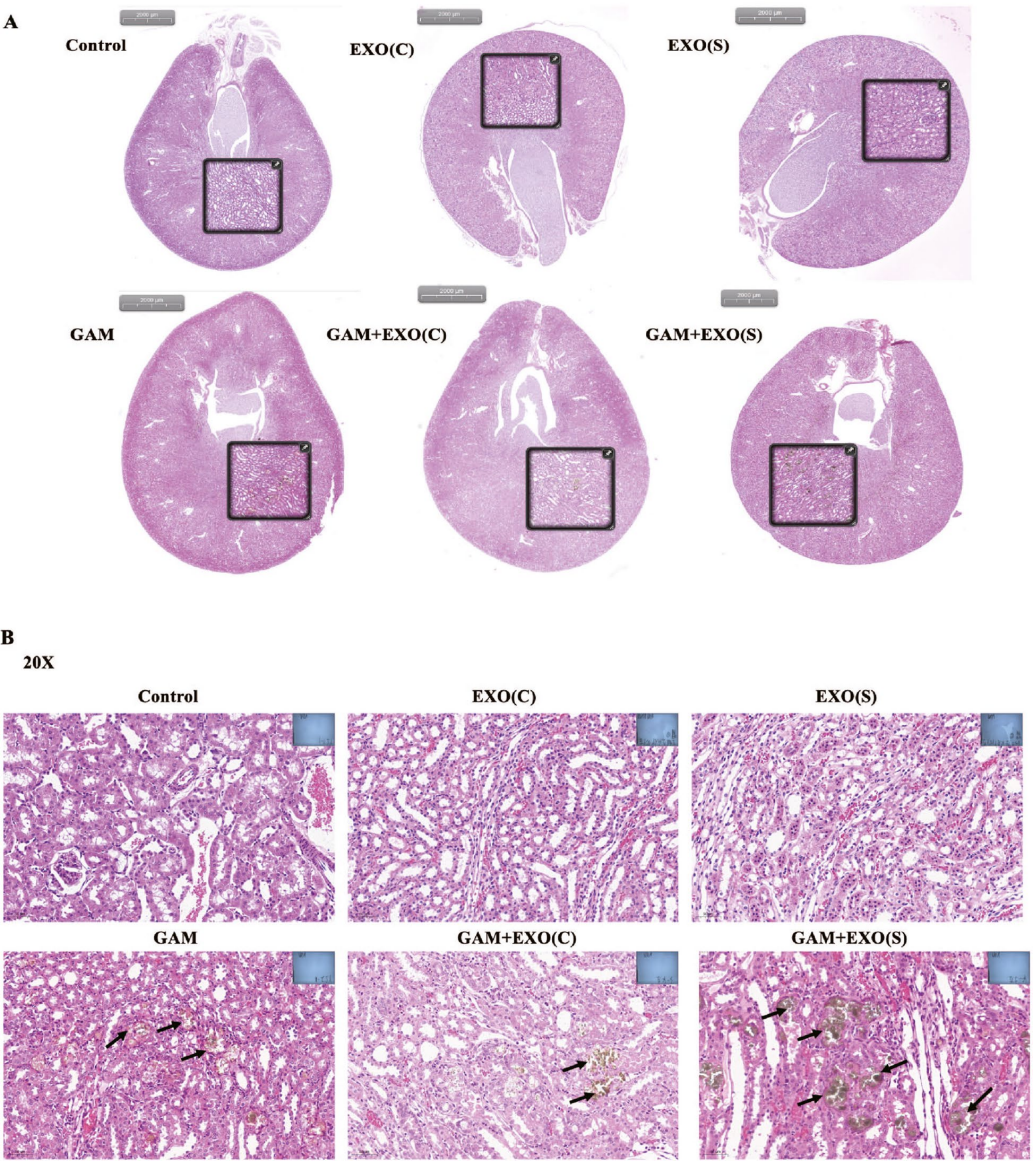
Effects of exosomes derived from HK2 cells under different conditions on crystal deposition in rat kidney. **A** A panoramic view of crystal deposition in kidney tissues with different treatments via Von Kossa staining. **B** Von Kossa staining to detect crystal deposition in the rat kidneys (magnification 200X). Black arrows indicate crystals

Preinjection of exosomes into SD rats with or without GAM stimulation was used to assess the effects of exosomes derived from HK2 cells under different conditions on crystal deposition in vivo. As shown in Fig. 8, Injection with only EXO(C) and EXO(S) did not result in a significant difference. Injection with GAM could accelerate crystal deposition, while preinjected with EXO(C) alleviated crystal deposition and preinjection with EXO(S) accelerated crystal deposition compared with that of the SD rats injected only with GAM (Fig. 8).

## Discussion

The prevalence of kidney stones has increased over the past years. Recurrent episodes and treatments result in a major burden on individuals and society. Therefore, it’s important to explore the mechanism of stone formation. Khan et al. indicated that inflammation and osteogenic changes play important roles in stone formation [14]. Previous studies have often focused on how CaOx crystals induced injury to the renal tubular epithelium [15]. However, the formation of Randall’s plaques is a lengthy and complicated process, and local interaction between CaOx crystals and epithelail cells is not sufficient for Randall’s plaque formation. The mechanism underlying prolonged and extended the effects of CaOx crystals, which induce cell injury and osteogenic changes, has become a problem that urgently needs to be addressed.

Intercellular crosstalk has been found to be important in many diseases, and an increasing number of recent studies have explored intercellular crosstalk [16], Hui li et al. found that intercellular crosstalk could modulate the formation of hepatocellular carcinoma [17], and Ondrej Kodet et al.found that intercellular crosstalk played important roles in the cancer microenvironment [18]. However, there are few studies on kidney stones. Extracellular vesicles have been confirmed to be important mediators of intercellular crosstalk. Increasing evidence has indicated that extracellular vesicles can mediate the bidirectional transfer of functional molecules (such as proteins, RNA and lipids) between cells [19]. Exosomes are a special type of extracellular vesicles with a diameter of 30-150 nm. These vesicles can mediate cell communication and signal transduction between cells because they package RNAs, proteins and many molecules that can modulate pathophysiological processes. Previous studies have confirmed that exosomes play important roles in many diseases; they can regulate the process of inflammatory bowel disease [20] and induce cancer development, metastasis and immunity [21]. Some studies have also focused on the roles of exosomes in CaOx stone formatiom. Nilubon Singhto et al. found that exosomes derived from macrophages stimulated with CaOx could enhance phagocytic activity of macrophages, which prevented stone formation [22]. Lei Yan et al. found that exosomes derived from calcium oxalate-treated macrophages promote apoptosis of HK-2 cells [23]. Injury to renal tubular epithelial cells has been suggested to initiatein the formation of CaOx stones [3]. However, to our knowledge, most studies on intercellular crosstalk have been limited to exosomes derived from macrophages, and there are no studies exploring exosomes derived from epithelial cells. We found that exosomes derived from HK2 cells could ameliorate crystal depositions induced by GAM, while exosomes derived from HK2 cells stimulated with CaOx crystals enhanced these effects. To explore the mechanism, we conducted experiments both in vivo and in vitro to gain new insight into the prevention and medical treatment of CaOx stones. Our study first confirmed that exosome-mediated crosstalk between epithelial cells could aggravate the cell injury cascade in CaOx stone formation.

In routine exosome studies, tail vein injection of exosomes was used for animal modelling. However, there is a relatively less aggregation of exosomes in targeted organs by using this approach. To solve this problem, we conducted subcapsular kidney injection to better allow exosomes to act on rat kidneys in our study. We found the duration of exosomes in rat kidneys was 1 week, so we injected exosomes at Days 1 and 7. As in vivo fluorescence imaging showed that the metabolic cycle of exosomes in rat kidneys was a week, we established the animal model for 2 weeks to ensure that the exosomes injected into the kidneys were fully used. Changes in animal modelling have led to enrichment of exosomes in kidneys, which improved the accuracy of animal models.

An increasing number of studies have suggested that oxidative stress and ROS are involved in renal epithelial injury and CaOx stone formation [24]. The level of renal ROS is significantly higher in patients with CaOx stones than in healthy controls [25]. Many studies have demonstrated that ROS can activate *MAPK* pathways [26]. *MAPK* cascades consist of *p-38, JNK* and *ERK* [27], and are involved in a wide ranfe of diseases processes [28]. Our previous studies have indicated that hypercalciuria and hyperoxaluria could promote the oxidative stress and active the *MAPK/P38* axis [6]. In our current study, we found that exosomes derived from HK2 cells stimulated with CaOx crystals (EXO(S)) could promote oxidative stress both in vivo and in vitro while EXO(C) did not result in any difference. Moreover, the expression of the *MAPK/P-38* pathway components was significantly activated with the stimulation of EXO(S), and preinjection of EXO(C) could reverse the effects induced by GAM. These results indicated that exosome-mediated intercellular crosstalk could amplify oxidative stress and MAPK pathway cascades. The amplification of these effects would enlarge the lesions of cells and finally lead to Randall’s plaque formation [29].

Sunil et al. found that osteogenic-related proteins, including *BMP2, OPN* and *OCN*, might play important roles in CaOx stone formation [30]. Chandi et al. suggested that ROS production could mediate BMP2 gene transcription and osteoblast differentiation [31]. Feng et al. also found that activation of *MAPK/P-38* signalling pathway could influence osteogenesis-related expression [32]. Moreover, our previous study confirmed that elevated oxidative stress could active the *MAPK/P-38* signalling pathway and that the activated *MAPK/P-38* axis could promote the expression of osteogenic-related proteins [6]. In our current study, we found that EXO(S) could promote the expression of *BMP2,OPN* and *OCN* both in vivo and in vitro. Preinjection of EXO(S) accelerated these effects in the rat kidneys injected with GAM, while preinjection of EXO(C) ameliorated these effects. These results indicated that exosomes derived from the HK2 cells stimulated by CaOx (EXO(S)) could induce positive feedback of osteogenic changes in vivo, and more epithelial cells with osteogenic changes produced more calcifying vesicles production. Calcifying vesicles could promote collagen deposition and mineralization, which led to the formation of Randall’s plaques [33]. Moreover, EXO(C) was a protective factor for CaOx stones and could be explored as a potential treatment or prevention.

As a carrier inducing intercellular crosstalk, exosomes function through the molecules they package [34]. In our research, we found that exosome-mediated intercellular crosstalk could amplify the oxidative stress and osteogenic change cascades of epithelial cells, which led to Randall’s plaque formation. However, the molecules packaged in these exosomes leading to these effects remain unclear. Our previous study found that *S100A8* and *S100A9* were highly expressed in exosomes derived from the urine of patients with CaOx stones, and they might play some role in stone formation [35]. We also found that *miR-223-3p* was significantly overexpressed in exosomes derived from HK2 cells stimulated with CaOx stones [13]. Therefore, exosomes derived from cells might be used as therapeutics for kidney stone disease or explored as new biomarkers. The core molecules packaged in exosomes inducing the amplification cascades of epithelial cell osteogenic changes would be further explored.

This study has certain limitations. First, this experiment was limited to in vitro and animal models, and the role of exosomes in humans remains to be explored. Second, our current study was just a preliminary exploration of the roles that exosomes might play in CaOx stone formation, and the core specific molecules packaged in the exosomes that led to CaOx stones still need further exploration.

## Conclusion

This study revealed that exosome-mediated intercellular crosstalk amplified epithelial cell injury cascades, which led to Randall’s plaque formation. EXO(S) could aggravate the formation of Randall’s plaques by promoting osteogenic changes through the *MAPK/P-38* axis. Moreover, EXO(C) ameliorate d the effects of GAM on rat kidneys, which indicated that EXO(C) may be a potential prevention or treatment for CaOx stones.

## Supplementary Information

**Additional file 1: Supplementary Table 1.** Primers we used in RT-PCR.

**Additional file 2: Supplementary Figure 1.** Effects of operation on oxidative stress injury. The sham control group showed that exosomes, rather than the operation itself, were responsible for these effects.

## Data Availability

The datasets used and analyzed in this study are available from the corresponding author on reasonable request.

## Abbreviations

GAM: Glyoxylic acid
TEM: Transmission Electron Microscopy
NTA: Nanoparticle Tracking Analysis
ROS: Reactive oxygen species
EXO(C): Exosomes derived from normal HK2 cells
EXO(S): Exosomes derived from HK2 cells stimulated with CaOx crystals
