# Supplementary material for: Exosome-mediated crosstalk between epithelial cells amplifies the cell injury cascade in CaOx stone formation
Source: J Biol Eng. 2023 Feb 28;17:16. doi: 10.1186/s13036-023-00324-0 (PMC9976448; doi:10.1186/s13036-023-00324-0)
Supplement: Supplementary file 1 — Additional file 1: Supplementary Table 1. Primers we used in RT-PCR. [file 13036_2023_324_MOESM1_ESM.docx]

Supplementary table1.Primers we used in RT-PCR.

| **Gene** | **Primer** | **Sequence (5'-3')** | **PCR Products** |
| --- | --- | --- | --- |
| b-actin | Forward | CACGATGGAGGGGCCGGACTCATC | 240bp |
|  | Reverse | TAAAGACCTCTATGCCAACACAGT |  |
| Homo BMP2 | Forward | TGCACCAAGATGAACACAGC | 230bp |
|  | Reverse | GTGGCAGTAAAAGGCGTGAT |  |
| Homo OPN | Forward | ACTGATTTTCCCACGGACCT | 192bp |
|  | Reverse | CTCCTCGCTTTCCATGTGTG |  |
| Homo OCN | Forward | CCTCACACTCCTCGCCCTAT | 132bp |
|  | Reverse | TTCACTACCTCGCTGCCCTC |  |
