# Supplementary figures and images for: Exosome-mediated crosstalk between epithelial cells amplifies the cell injury cascade in CaOx stone formation
Source: J Biol Eng. 2023 Feb 28;17:16. doi: 10.1186/s13036-023-00324-0 (PMC9976448; doi:10.1186/s13036-023-00324-0)

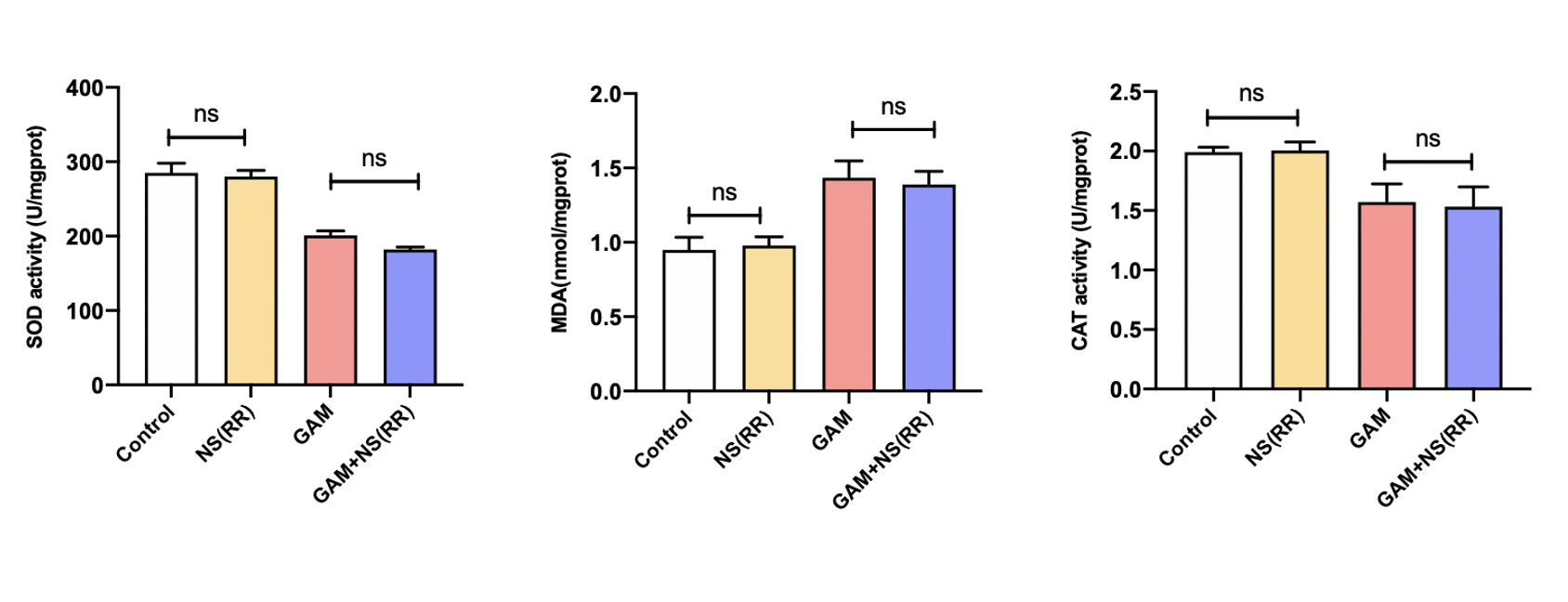

Supplement: Supplementary file 2 — Additional file 2: Supplementary Figure 1. Effects of operation on oxidative stress injury. The sham control group showed that exosomes, rather than the operation itself, were responsible for these effects. [file 13036_2023_324_MOESM2_ESM.png]
